# The influence of weather on the population dynamics of common mosquito vector species in the Canadian Prairies

**DOI:** 10.1186/s13071-023-05760-x

**Published:** 2023-04-28

**Authors:** Cole Baril, Ben G. Pilling, Milah J. Mikkelsen, Jessica M. Sparrow, Carlyn A. M. Duncan, Cody W. Koloski, Stefanie E. LaZerte, Bryan J. Cassone

**Affiliations:** 1 Department of Biology, Brandon University, Brandon, MB R7A 6A9 Canada; 2 Steffi LaZerte R Programming and Biological Consulting, Brandon, MB Canada

**Keywords:** Temperature, Humidity, Rainfall, *Culex*, *Aedes*, GLMM

## Abstract

**Background:**

Mosquito seasonal activity is largely driven by weather conditions, most notably temperature, precipitation, and relative humidity. The extent by which these weather variables influence activity is intertwined with the animal’s biology and may differ by species. For mosquito vectors, changes in weather can also alter host–pathogen interactions thereby increasing or decreasing the burden of disease.

**Methods:**

In this study, we performed weekly mosquito surveillance throughout the active season over a 2-year period in Manitoba, Canada. We then used Generalized Linear Mixed Models (GLMMs) to explore the relationships between weather variables over the preceding 2 weeks and mosquito trap counts for four of the most prevalent vector species in this region: *Oc. dorsalis*, *Ae. vexans*, *Cx. tarsalis*, and *Cq. perturbans*.

**Results:**

More than 265,000 mosquitoes were collected from 17 sampling sites throughout Manitoba in 2020 and 2021, with *Ae. vexans* the most commonly collected species followed by *Cx. tarsalis*. *Aedes vexans* favored high humidity, intermediate degree days, and low precipitation. *Coquillettidia perturbans* and *Oc. dorsalis* activity increased with high humidity and high rainfall, respectively. *Culex tarsalis* favored high degree days, with the relationship between number of mosquitoes captured and precipitation showing contrasting patterns between years. Minimum trapping temperature only impacted *Ae. vexans* and *Cq. perturbans* trap counts.

**Conclusions:**

The activity of all four mosquito vectors was affected by weather conditions recorded in the 2 weeks prior to trapping, with each species favoring different conditions. Although some research has been done to explore the relationships between temperature/precipitation and *Cx. tarsalis* in the Canadian Prairies, to our knowledge this is the first study to investigate other commonly found vector species in this region. Overall, this study highlights how varying weather conditions can impact mosquito activity and in turn species-specific vector potential.

**Graphical Abstract:**

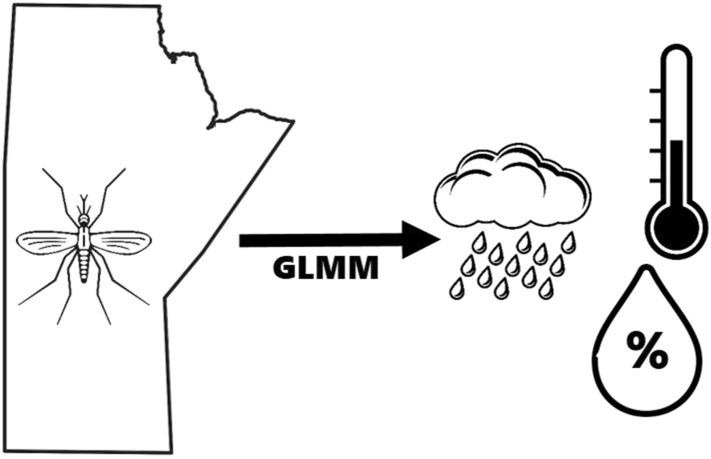

**Supplementary Information:**

The online version contains supplementary material available at 10.1186/s13071-023-05760-x.

## Background

Global climate change is widespread and affects zoonoses by increasing (1) the geographical range and/or pervasiveness of animal reservoirs and/or arthropod vectors; (2) introductions of competent vectors, and/or the occurrence, intensity; and (3) the duration of transmission cycles [1]. Canada continues to show signs of a changing climate, including a ~ 2 °C rise in annual surface temperatures since 1948 [2]. The Intergovernmental Panel on Climate Change (IPPC, 2018) has indicated that mosquito-borne diseases (MBDs) will be particularly impacted by climate change. Indeed, seasonal variations in the occurrence and abundance of mosquito populations are intricately tied to climatic factors, which in turn impact their vector potential [3]. In Canada, the burden of MBDs has increased by 10% in the past 20 years and is expected to continue to rise if the impacts of climate change are not mitigated [4].

Several studies have shown strong associations between mosquito vector abundances and weather factors [5–8]. Temperature, precipitation, and relative humidity are the three major weather variables influencing mosquito seasonal activity [9–12] and host–pathogen interactions [13]. Temperature can impact mosquito survival [14–16], development [4, 16, 17], geographical range [18, 19], vector competence [20–22], and host-seeking and other behaviors [23]. Precipitation can alter the occurrence of suitable larval habitats [22, 24, 25] and the viability of eggs and larvae [26]. Humidity can affect the mating, dispersal, longevity, bloodfeeding behaviors, and oviposition of mosquitoes [3, 27, 28]. However, there are numerous mitigating factors that can drastically alter mosquito population dynamics (e.g., forest cover), and the combined effects of multiple abiotic and biotic factors are often challenging to resolve.

Mosquitoes typically have species-specific ranges of weather conditions for optimal seasonal activity. Higher temperatures are generally favored by mosquitoes [29], but species can vary in their minimum thresholds. For instance, the minimum metabolic threshold for *Aedes vexans* is 12 °C, but is slightly lower (10 °C) for *Culex tarsalis* and *Coquillettidia perturbans* [30–32]. To this end, some studies associate mosquito abundance with degree days, which is a weather-based indicator that takes into account both ambient temperatures and minimum metabolic thresholds of a given species [30, 33]. Standing water from rainfall creates necessary breeding grounds for many species, but too much precipitation can wash away larval habitats [34–36]. Thus, species that utilize more permanent breeding grounds (e.g., lakes, marshes) are likely less susceptible to population fluctuations associated with rainfall. The overall relationship between mosquito abundance and precipitation is not straightforward, however, as some species abundances appear most influenced by rainfall occurring weeks to even months prior [5, 30, 37]. In contrast, high humidity conditions are typically preferred by mosquitoes, as sustained bouts of low moisture can impact their survival, behaviors, and development [38].

There are several species of mosquitoes found in the Canadian Prairies that can potentially harbor and transmit viruses of public health concern. *Aedes vexans* Meigen, the inland floodwater mosquito, is a cosmopolitan nuisance mosquito with broad vector potential. It is capable of transmitting West Nile virus (WNV), California serogroup viruses (CSGVs), Rift Valley fever virus, and Zika virus [39–42]. *Ochlerotatus dorsalis* Meigen, the summer saltmarsh mosquito, is found throughout North America and is a competent vector of Western equine encephalitis virus (WEEV), CSGVs, and WNV [43]. The cattail mosquito, *Coquillettidia perturbans* Walker, is found throughout the Prairies, breeding in permanent swamps containing cattails and aquatic plants [43]. This species is associated with the transmission of Eastern equine encephalitis virus (EEEV), WNV, and CSGVs [43, 44]. The geographical range of *Culex tarsalis* Coquillett extends from northern Mexico into Canada and from the west coast to the Mississippi River [45, 46]. The species is the primary vector of WNV in the Prairies and also capable of transmitting WEEV and CSGVs [43, 47]. Other mosquito vector species occurring in the Prairies include *Aedes canadensis* (CSGVs, WNV, EEEV), *Ochlerotatus triseriatus* (La Crosse virus, EEEV, WEEV), and *Ochlerotatus trivittatus* (CSGVs) [19, 43, 47–49]. Although these vectors presumably have varying optimal ranges for temperature, humidity, and precipitation, little information is presently available on the relationships between weather factors and mosquito seasonal activity in the Canadian Prairies.

Two Canadian Prairie provinces (Manitoba and Saskatchewan) carry out annual mosquito surveillance to detect the causal agents of MBDs at the provincial level. However, these programs focus their monitoring activities on *Culex* species capable of transmitting WNV, most notably *Cx. tarsalis*. To our knowledge no other mosquito vector species are identified or tested for human pathogens in these programs. Consequently, we carried out weekly surveillance during the active season over a 2-year period in Manitoba to characterize the population dynamics of nine commonly found mosquito species. We then used Generalized Linear Mixed Models to determine the relationships (if any) between mosquito trap counts and weather variables (temperature, precipitation, and relative humidity) for the four most abundant vector species. Since the life cycle (egg-to-adult) of most species is between 8 and 14 days, the combination of weather conditions over this period is likely to affect development and thus mosquito abundance and activity [32, 43]. Consequently, our analyses investigated how the 14 days preceding the trapping date impacted the number of mosquitoes captured.

## Material and methods

### Mosquito trapping and identification

Host-seeking mosquitoes were trapped using CDC Miniature Light Traps (Model 1012, John W. Hock, Gainesville, FL) with carbon dioxide (CO_2_) regulators set to 15 psi and the light disabled (to minimize non-mosquito collections). We placed traps on tree limbs ~ 1.5 m from the ground and activated them from dusk until dawn. Traps were operated twice weekly (Monday and Tuesday) in 2020 and 2021, from June to August (CDC weeks 23 to 36). A total of 24 traps were deployed in eight Western Manitoba communities in 2020, with one trap setup in each community in 2021 (Additional files 1 and 2). In 2020, collections from one-time satellite traps from nine additional locations in Central and Eastern Manitoba were provided to us by the City of Winnipeg Insect Control Branch with *Culex* species removed (Additional files 1 and 2). All mosquitoes were stored at − 80 °C in Petri dishes coded by date and collection site.

Five mosquito vector species were visually identified using dissecting microscopes in 2020: *Ochlerotatus flavescens* Muller, *Oc. dorsalis*, *Ae. vexans*, *Cx. tarsalis*, and *Cq. perturbans*. We expanded our identification efforts to include four less common and/or non-vector species in 2021: *Aedes canadensis* Thebald, *Ochlerotatus trivittatus* Coquillett, *Ochlerotatus triseriatus* Say, and *Anopheles earlei* Vargus. Specimens were identified to species using relevant mosquito identification keys [43, 50, 51]. For traps with high numbers of specimens (> 1000), we subsampled by counting a randomized ¼ sample of the trap and then extrapolated the numbers by a factor of four.

### Weather factors associated with mosquito counts

For each trapping location over the 2-year surveillance period, we recorded three variables that may be connected to mosquito trap catch: temperature (°C), precipitation (mm), and relative humidity (%). These data were obtained from the Environment Canada weather station closest to each trapping location. Weather data was collected daily from each location between May and August in both years. The distances between trapping site and the closest servicing weather station ranged from < 1 to 63 km. The reason some of the stations are farther away than others is they service multiple towns that have had historically comparable weather indices (Environment Canada, personal communication).

We focused our analyses (see below) on the four most commonly found vector species: *Oc. dorsalis*, *Ae. vexans*, *Cx. tarsalis*, and *Cq. perturbans*. The specific variables explored were: (1) mean rainfall (mm) over the 14 days preceding the trapping date (ppm_14_); (2) mean relative humidity (%) 14 days prior to the trapping date (rhm_14_); and mean degree days 14 days preceding the trapping date (ddm_14_). The latter incorporated mean daily temperatures (*T*_mean_; °C) and baseline metabolic temperature (Tb; °C) for each mosquito species [30–32], where ddm_14_ represents the number of degrees above *T*_base_ over the 14 d period; thus if dd_1_: *T*_mean_ > *T*_base_, then dd_1_ = *T*_mean_ − *T*_base_, but if dd_1_: *T*_mean_ ≤ *T*_base_, then dd_1_ = 0 °C [30]. Since Tb is not published for *Oc. dorsalis*, we used the same value (12 °C) as *Ae. vexans*, which is also a floodwater species. Preliminary exploration suggested that trap count differences with local weather variation were species-specific. We therefore modelled weather variables separately for each species. Finally, as low temperatures can inhibit mosquito activity and therefore influence trap counts we included trapping day minimum temperature as a covariate to account for this effect.

### Statistical analyses

Relationships among environmental variables and mosquito counts over 2 years (2020 and 2021) were assessed for each species (*Oc. dorsalis*, *Ae. vexans*, *Cx. tarsalis*, and *Cq. perturbans*) using R statistical software (v4.2.1; [52]). We explored two sets of models: (1) a single model exploring the effect of mosquito species on trap counts; and (2) four species-specific models exploring the effects of time (CDC week) and weather variables (ppm_14_, rhm_14_, ddm_14_) on trap counts. All models were Generalized Linear Mixed Models (GLMMs; glmmTMB package v1.1.2.3; [53]). To control for spatial, site-level effects we included trap location nested within site as a random intercept. To control for temporal, week-level effects we also included a categorical week-by-year variable as a random intercept. We used a negative binomial distribution because while we have count data, they were over-dispersed and did not match a Poisson distribution.

For the species model, trap counts were modelled using the complete data set with species, year, and their interaction as explanatory variables. Each trap count was represented by a row in the data with no pooling of counts within or between weeks/sites. Minimum temperature of the trap-day was included as a covariate. Because this full dataset showed significant temporal autocorrelation, we added an AR(1) covariance structure to week grouped by unique site (across years). We conducted Post-Hoc analyses to compare species differences among years (emmeans package v1.7.0; [54]) with the false discovery rate *P*-value adjustment.

For the weather models, curvilinear relationships in the weather variables and week were modelled as 2nd degree orthogonal polynomials. We also included interactions between year and each polynomial: trapcount ~ poly(week, degree = 2) * year + poly(ddm14, degree = 2) * year + poly(pt14, degree = 2) * year + poly(rha14, degree = 2) * year + ttmin + (1|week_year) + (1|site/sitespecific). Further, we included minimum temperature of the trap-day as a covariate. Where non-significant (alpha of 5%), individual polynomial terms and interactions were omitted from the models and linear terms and main effects retained alone. Type III ANOVA tables were computed with the car package (v3.0.12; [55]) and used to assess polynomial terms. Significant linear effects were additionally reported with summary table statistics (Estimate and *Z*-test) in order to capture the magnitude of the effect (i.e. the Estimate). To aid in the interpretation we converted the original estimates to incident rate ratios which can be interpreted as a multiplicative factor (i.e. an incident rate ratio of 2 indicates a 2 times increase).

All model fits and assumptions (including potential spatial and temporal autocorrelation) were assessed with the DHARMa package (v0.4.4; [56]); multicollinearity was assessed with the performance package (v0.8.0; [57]); and figures were created with the ggplot2 package (v3.3.5; [58]). Note that figure scales are log_10_ transformed after first adding 1 to better visualize patterns.

## Results

### Mosquito surveillance activities

More than 265,000 mosquitoes were collected throughout southern Manitoba over the 2-year surveillance period, with 57% captured in 2020. This included weekly collections in Western Manitoba and one-time satellite collections at various times between June and September in Eastern and Central Manitoba, though the latter represented a small proportion (11%) of the total mosquito catch. Trap counts tended to be highest between weeks 26 and 29, though this differed to some extent by species and year. Notable were fogging events in Brandon in both 2020 and 2021, which subsequently resulted in considerably reduced mosquito numbers in that community. Consequently, we omitted mosquito trap count data from this site post-fogging for all analyses (CDC weeks ≥ 30 in 2020, weeks ≥ 28 in 2021).

### *Aedes vexans* was the most common mosquito species

**Fig. 1:**
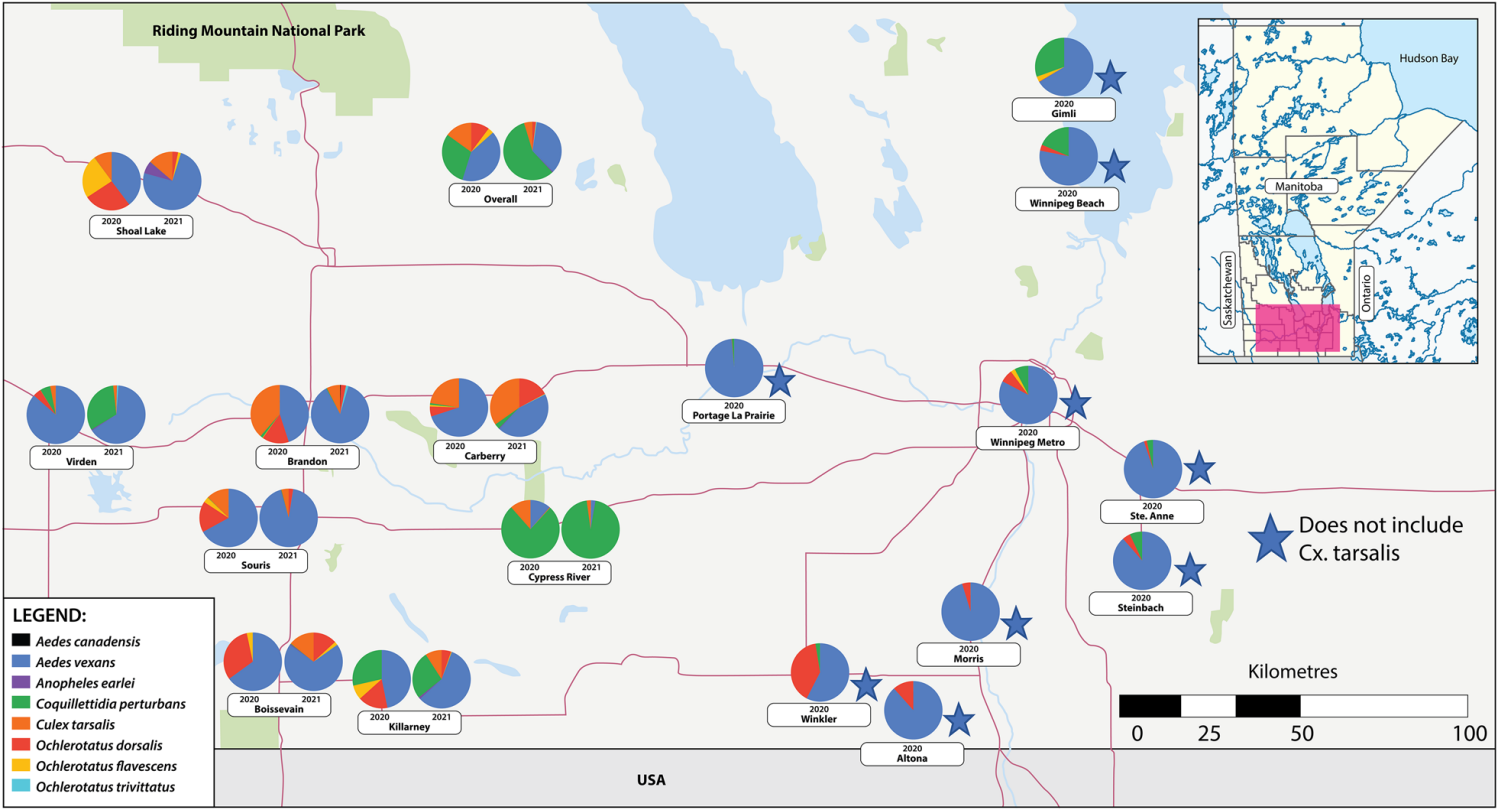
Relative trap counts for the eight most commonly found mosquito species in 2020 and 2021. Mosquitoes were captured on a weekly basis (May to September) from 17 sampling sites throughout Manitoba, Canada. *Culex tarsalis* counts are not included for all locations in the eastern part of the region (denoted with an asterisk*). *Ae. canadensis*, *An. earlei*, *Oc. trivittatus*, and *Oc. triseriatus* were not surveyed in 2020. We collected one *Oc. triseriatus* in 2021, which was not included on the figure

**Fig. 2:**
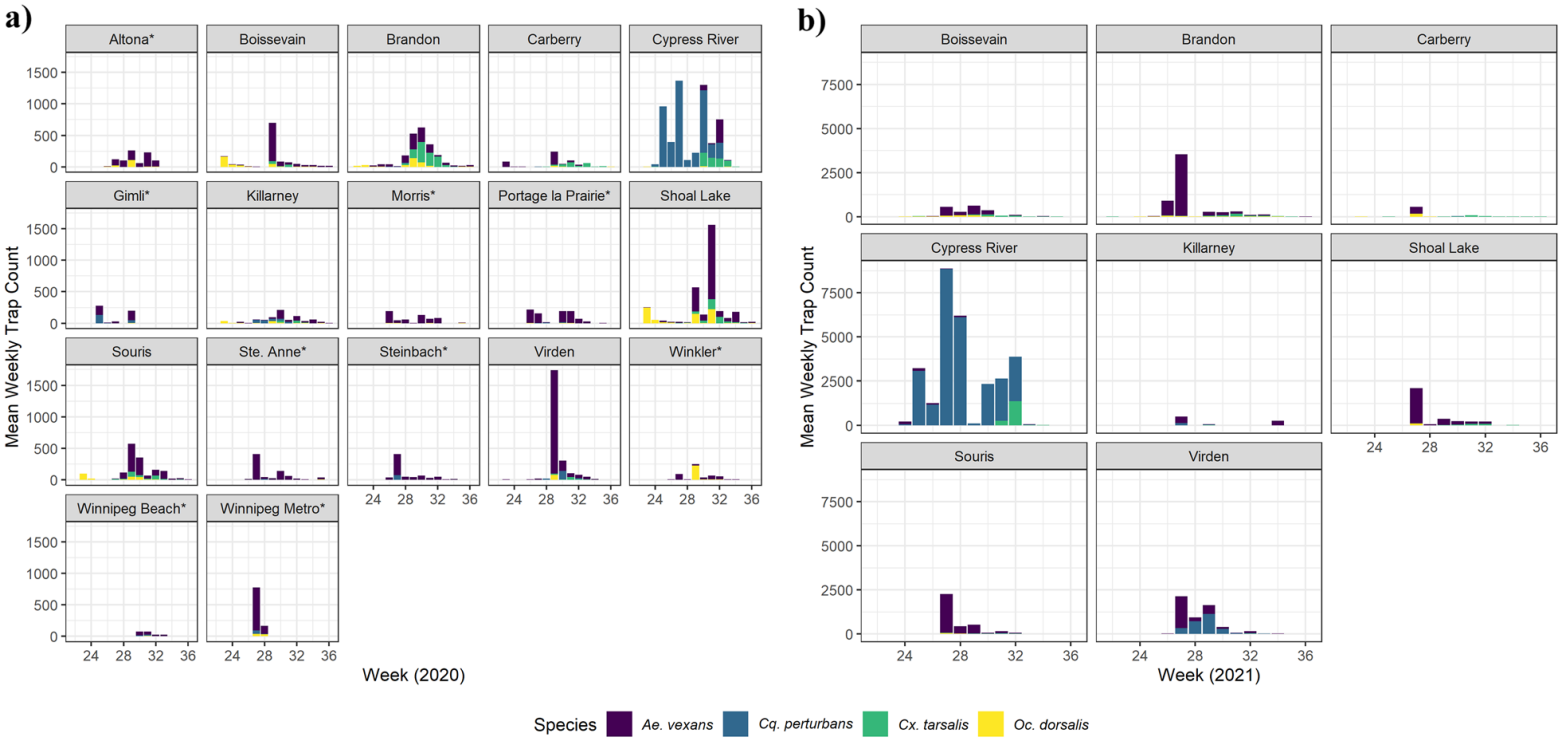
Average weekly trap counts for each of the 17 sampling sites in 2020 **a** and 2021 **b**. Displayed are the four most commonly collected vector species: *Ae. vexans, Cq. perturbans, Cx. tarsalis*, and *Oc. dorsalis*. *Culex tarsalis* counts are not included for all locations in the eastern part of the region (denoted with an asterisk*)

There was considerable variation in the relative proportions of each mosquito species per trapping location (Fig. 1), as well as over time (Fig. 2). Of the mosquitoes caught, 40% (2020) and 80% (2021) represented the four primary vector species: *Ae. vexans, Oc. dorsalis, Cx. tarsalis*, and *Cq. perturbans*. This discrepancy in proportions between years is largely attributed to *Cq. perturbans* from a single location (Cypress River), where > 50,000 individuals were captured in 2021 and only 14,000 in 2020. As this site reflected drastically and systematically different trapping patterns (Figs. 1, 2) we removed the site from all subsequent analyses (outlier effect).

There was a significant interaction between species and year (*χ*^2^_3_ = 33.57; *P* < 0.0001) and we therefore conducted Post-Hoc analyses separately for each year. These showed that in both years, we captured significantly more *Ae. vexans* than each of the three other primary vector species (*P* < 0.01 across all pairwise comparisons;). *Ochlerotatus dorsalis* trap counts were 2.4× higher than *Cx. tarsalis* in 2020 (*P* < 0.001) but 0.28× lower in 2021 (*P* < 0.001). However, both *O. dorsalis* and *Cx. tarsalis* had higher trap counts than *Cq. perturbans* in 2020 (9.3× and 3.4×; both *P* < 0.0001) as well as in 2021 (2.5× and 9.0×; both *P* < 0.05).

### The influence of weather variables on trap counts was species-specific

#### Aedes vexans

**Fig. 3:**
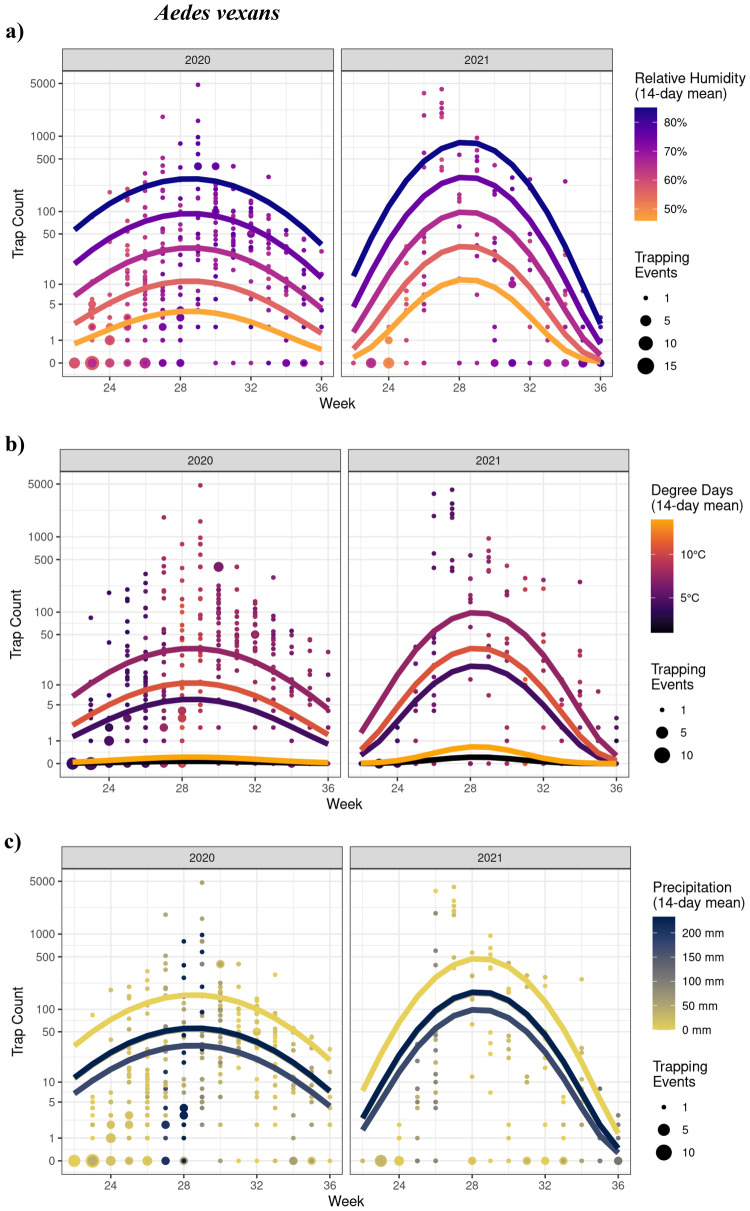
GLMM model analyses showing the effects of time (CDC week) and weather variables on *Aedes vexans* trap counts in 2020 and 2021. Seasonal mosquito activity was significantly impacted by **a** relative humidity, **b** degree days, and **c** precipitation in the 2-week period preceding the trapping date. Week corresponds to the week of the year for 2020 and 2021 (e.g., week 24 is the 24th week of both 2020 and 2021). Points represent observed data

There was a significant interaction between year and the 2nd order polynomial for trap week (*χ*^2^_2_ = 6.34; *P* = 0.042). As such, trap counts increased and then decreased over the season (with a greater increase in 2021) (Fig. 3). Trap counts increased linearly with relative humidity (*χ*^2^_1_ = 9.35; *P* = 0.002), with 1.12× (12%) more mosquitoes captured for every % increase in relative humidity (Est = 0.109; *z* = 3.057; Fig. 3a). In addition, there were significant quadratic effects of degree days (*χ*^2^_2_ = 19.58; *P* < 0.0001) and precipitation (*χ*^2^_2_ = 11.03; *P* = 0.004). Accordingly, trap counts were highest with intermediate values of degree days (Fig. 3b) and low precipitation (Fig. 3c). Trapping day minimum temperature had a significant effect (*χ*^2^_1_ = 5.13; *P* = 0.024), where trap counts were 1.07× (7%) greater with every 1 °C increase in minimum temperature (Est = 0.072; *z* = 2.265). Notably the model fit (assessed by checking patterns in the residuals) for this species was marginal, suggesting other factors may be in play that were not been captured by this model.

#### Culex tarsalis

**Fig. 4:**
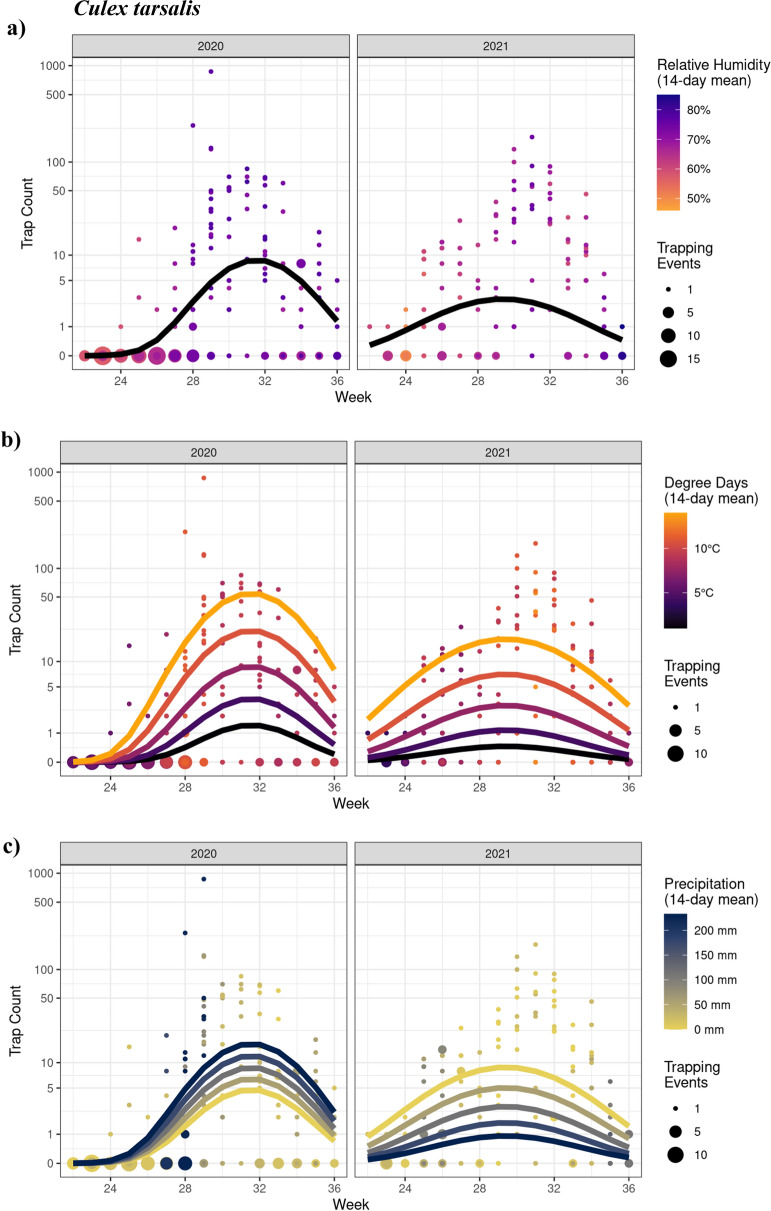
GLMM model analyses showing the effects of time (CDC week) and weather variables on *Culex tarsalis* trap counts in 2020 and 2021. Seasonal mosquito activity was significantly affected by **b** degree days and **c** precipitation in the 2-week period preceding the trapping date. Since there was no significant effect of **a** relative humidity, only one, black, line is shown (seasonal effect). Week corresponds to the week of the year for 2020 and 2021 (e.g., week 24 is the 24th week of both 2020 and 2021). Points represent observed data

There was a significant interaction between year and the 2nd order polynomial for trap week (*χ*^2^_2_ = 16.17; *P* = 0.0003), such that trap counts increased and then decreased over the season with a more distinct peak in 2020 (Fig. 4). There were no associations (quadratic or linear) between trap counts and relative humidity (*χ*^2^_1_ = 2.52; *P* = 0.112; Fig. 4a). However, there was a linear relationship between trap counts and degree days (*χ*^2^_1_ = 4.16; *P* = 0.041; Fig. 4b), such that the number of mosquitoes captured increased by 32% for every 1 °C increase in mean degree days (Est = 0.281; *z* = 2.040). There was a significant interaction between year and precipitation (*χ*^2^_1_ = 4.73; *P* = 0.030). Accordingly, the relationship between trap counts and rainfall showed contrasting patterns, with a greater number of mosquitoes captured with high and low precipitation in 2020 and 2021, respectively (Fig. 4c). There was no effect of trapping day minimum temperature on trap counts (*χ*^2^_1_ = 2.05; *P* = 0.152).

#### Coquillettidia perturbans

**Fig. 5:**
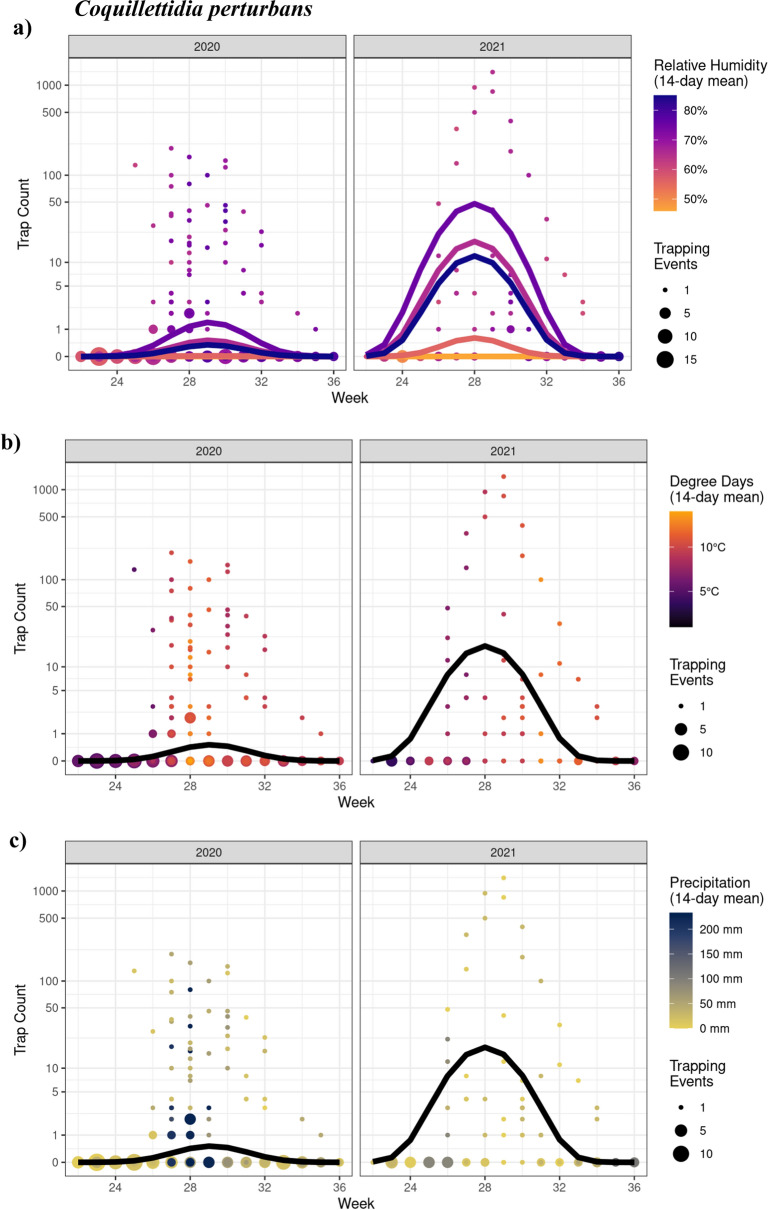
GLMM model analyses showing the effects of time (CDC week) and weather variables on *Coquillettidia perturbans* trap counts in 2020 and 2021. Seasonal mosquito activity was significantly influenced by **a** relative humidity in the 2-week period preceding the trapping date. Since there was no significant effect of **b** degree days or **c** precipitation, only one, black, line is shown (seasonal effect). Week corresponds to the week of the year for 2020 and 2021 (e.g., week 24 is the 24th week of both 2020 and 2021). Points represent observed data

There was a significant interaction between year and the 2nd order polynomial for trap week (*χ*^2^_*x*_ = 7.74; *P* = 0.0209), such that trap counts increased and then decreased over the season (with a relatively more consistent peak in 2021) (Fig. 5). We also identified a significant quadratic relationship between trap counts and relative humidity (*χ*^2^_2_ = 9.10; *P* = 0.011), with elevated (but not extreme) humidity resulting in a greater number of mosquitoes captured (Fig. 5a). There were no effects (quadratic or linear) of degree days (*χ*^2^_1_ = 0.81; *P* = 0.368; Fig. 5b) nor precipitation (*χ*^2^_1_ = 0.24; *P* = 0.622; Fig. 5c). Trapping day minimum temperature had a significant effect (*χ*^2^_1_ = 29.62; *P* < 0.0001), where trap counts were 1.43 times greater with every 1 °C increase in minimum temperature (Est = 0.36; *z* = 5.44).

#### Ochlerotatus dorsalis

**Fig. 6:**
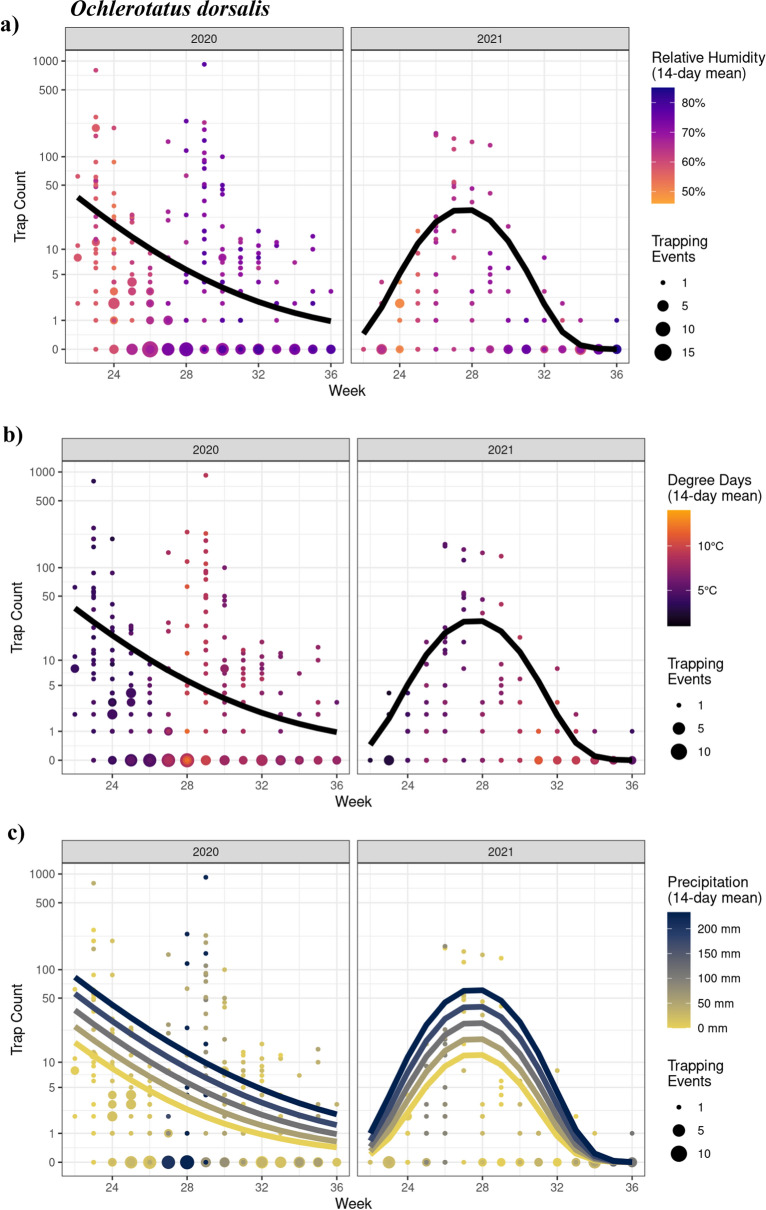
GLMM model analyses showing the effects of time (CDC week) and weather variables on *Ochlerotatus dorsalis* trap counts in 2020 and 2021. Seasonal mosquito activity was significantly impacted by **c** precipitation in the two-week period preceding the trapping date. Since there was no significant effect of **a** relative humidity and **b** degree days, only one, black, line is shown (seasonal effect). Week corresponds to the week of the year for 2020 and 2021 (e.g., week 24 is the 24th week of both 2020 and 2021). Points represent observed data

There was a significant interaction between year and the 2nd order polynomial for trap week (*χ*^2^_2_ = 15.94; *P* < 0.001). As such, trap counts in 2020 decreased almost linearly, but in 2021 showed a pattern of increased and decreased numbers over the season. The was no effect (linear or quadratic) of relative humidity (*χ*^2^_1_ = 1.67; *P* = 0.196; Fig. 6a) nor degree days (*χ*^2^_1_ = 0.47; *P* = 0.494; Fig. 6b) on trap counts. However, we identified a significant linear relationship between trap counts and precipitation (*χ*^2^_1_ = 3.89; *P* = 0.049; Fig. 6c). Accordingly, trap counts increased by 1.01 times (1%) for each mm increase in precipitation (Est = 0.0069; *z* = 1.972). There was no effect of minimum trapping day temperature (*χ*^2^_1_ = 0.21; *P* = 0.646).

## Discussion

The primary objective of our study was to explore the relationships between key weather variables and mosquito population dynamics in the Canadian Prairies. Our two consecutive years of weekly trapping throughout southern Manitoba yielded over 265,000 mosquitoes, of which the majority represented four noted vector species: *Oc. dorsalis*, *Ae. vexans*, *Cx. tarsalis*, and *Cq. perturbans*. It should be emphasized that the trap counts provide a good indication of mosquito activity (i.e., host-seeking) during the trapping period, but do not necessarily correlate with overall mosquito abundances at a given sampling site. Further, we discuss weather conditions favored by mosquitoes as it relates to higher trap counts rather than their true abundance/activity/biology. *Aedes vexans* was the most common mosquito in most sites/weeks, which is in agreement with historical records for our sampling region [59] and nearby regions [41, 47]. Both *Oc. dorsalis* and *Cx. tarsalis* are also well established in the Canadian Prairies [60, 61]. Interestingly, *Cq. perturbans* trap counts were relatively low with the exception of one site, Cypress River. This is likely due to habitat suitability, as the larvae of this species feed on cattails [43, 62, 63] and our traps at Cypress River were situated adjacent a marsh-like area with heavy aquatic vegetation that included abundant cattails. Consequently, *Cq. perturbans* activity at this site appears driven by breeding site conditions rather than weather variables.

In terms of seasonal activity, *Ae. vexans*, *Cx. tarsalis*, and *Cq. perturbans* all showed a similar (and expected) pattern, with trap counts gradually increasing to peak numbers before progressively declining. However, the peak in trap counts occurred later in the season for *Cx. tarsalis* (late-July to early August) in comparison to the other two species (early- to mid-July). This discrepancy is likely attributed to the overwintering behaviors of these species. While *Cx. tarsalis* overwinter as non-fed adults, *Ae. vexans* and *Cq. perturbans* overwinter in the egg stage and as larvae, respectively [31, 43, 64, 65]. Consequently, the former requires a bloodmeal prior to laying eggs thereby delaying the first generation in comparison to the other two species. Trap counts for all three species were higher in 2021 compared to 2020, presumably due to more favorable environmental conditions for adult survival, oviposition success, and/or host-seeking activities. Although *Oc. dorsalis* showed a seasonal trend similar to the other mosquito species in 2021, trap counts in the previous year were highest at or near the start of our surveillance activities. This suggests an early spring emergence of *Oc. dorsalis* in 2020, which is consistent with their known biology [66]. However, the steady decline in their numbers throughout the 2020 season was unexpected given *Oc. dorsalis* can have multiple generations per year [67]. This suggests some combination of environmental factors later in the season and comparatively low sample sizes may have impeded the success of subsequent generations.

Mosquito seasonal activity is largely driven by temperature, precipitation, and relative humidity [9–12]. Given most species complete development within 14 days, we focused on how temperature, precipitation, and relative humidity in the two weeks preceding the trapping date affected mosquito counts. Both *Ae. vexans* and *Cq. perturbans* favored (i.e., higher trap counts) high humidity (75–85%), which is consistent with studies from other geographic regions [23, 68]. High humidity has been associated with increased egg production, larval indices, adult survival and activity, including host-seeking at close range [9, 11, 26, 68, 69]. In contrast, bouts of low humidity can cause eggs to desiccate and reduce adult longevity and/or activity in favor of seeking shelter [27, 28, 70]. The lack of a relationship between *Cx. tarsalis* and *Oc. dorsalis* trap counts and humidity was a bit unexpected, though the biology of both species suggests that their activity is less impacted by humidity. Stuart (2020) found *Culex* mosquitoes to prefer breeding under hot and dry conditions [71] with adult *Cx. tarsalis* typically reared in laboratories under relatively low humidity [72]. *Ochlerotatus dorsalis* is found in a wide rage of habitats (e.g., coastal marshes, grasslands, forests, tidal areas, and semiarid deserts), which can vary considerably in relative humidity [73, 74].

Since mosquito development is temperature-dependent, their abundance typically increases with air temperature (i.e., faster lifecycle) and then declines once a threshold has been reached [32, 75]. This threshold varies depending on species, with reported ranges between 22 and 30 °C [32, 76–80]. Moreover, mosquitoes have minimum metabolic temperatures (e.g., [30–32]) for which their activity largely ceases below this level. Consequently, we focused only on the air temperatures fostering mosquito development over the 14 days preceding the trapping date. *Culex tarsalis* favored high degree days*,* which is consistent with their biology in the Canadian Prairies [61]. *Aedes vexans* favored intermediate degree days, presumably having increased mortality and/or reducing their activity by seeking refuge during bouts of extreme temperatures [81–83]. Temperature did not impact *Cq. perturbans* nor *Oc. dorsalis* trap counts, which may be attributed to the unique habitat requirements of the former and the diversity of suitable habitats for the latter. It is also possible that increasing the lag period to more than 14 days may better capture the effect of temperature on both species, as found for other species/regions [5, 30]. Finally, minimum trapping day temperature was associated with trap counts for *Ae. vexans* and *Cq. perturbans* but not the other three species. Temperatures between 15 and 24 °C are generally suitable for host-seeking activities in mosquitoes [23], but both species may have a narrower temperature range for optimal activity.

The influence of precipitation on mosquito life history traits is often complex and differs among species and studies [6, 30, 37, 84–87]. Even within a species the relationship is not always clear. For instance, some studies have found a positive effect of rainfall on *Ae. alpbopictus* abundance [88–91], whereas others have not [92, 93]. Although the larval stages of all mosquitoes are dependent on water availability, their breeding habitats, oviposition biology, and egg physiology can vary markedly. Indeed, rainfall over the 14 days prior to the trapping date influenced mosquito counts for *Ae. vexans*, *Cx. tarsalis*, and *Oc. dorsalis*, but the underlying biological reasoning was not always obvious. Larval breeding sites for *Oc. dorsalis* include temporary pools formed by precipitation [73], which is in line with the higher levels of rainfall that this species favors. However, *Ae. vexans* favoring dryer conditions was unexpected as this species oviposits on soil, relying on precipitation events to trigger egg hatching [94, 95]. Consequently, *Ae. vexans* should increase in abundance with higher levels of short-term rainfall. Precipitation influenced *Cx. tarsalis* activity, but the species favored higher and lower levels of rainfall in 2020 and 20,921, respectively. Given *Cx. tarsalis* and *Cq. perturbans* lay their eggs directly on the surface of water [96, 97], they are more likely to be influenced by longer-term precipitation. This would presumably dictate the number of suitable breeding sites available and in turn mosquito abundance several weeks later [37]. Indeed, abundance of some mosquito species has been positively associated with rainfall events occurring several weeks to even months later [5, 30, 37, 86].

There are several considerations related to experimental design that must be taken into account when forming conclusions from our study. The interactions between individual weather factors, additional factors not examined in this study (e.g., wind velocity, moonlight, and anthropogenic water sources), and their combined effects are nearly impossible to disentangle without controlled experiments. Some weather variables, particularly precipitation, may show improved modelling for some species with longer lag periods from the trapping date, though it would be challenging to infer the specific reason(s) for any significant relationships. The precision of our study could also be improved by setting up weather stations directly at each trapping site. Further, our traps predominately captured host-seeking females from dawn to dusk, with most traps set up on the interface between human dwellings and forest/agricultural land. Given the differences in host-seeking behaviors and ecologies among species, this design may not accurately reflect the true relative abundances of each species. Deploying multiple trap types (e.g., gravid, net, BG-Sentinel) to supplement our collections in conjunction with sampling sites with varying land cover (e.g., forest, urban and rural areas, agricultural areas) may better inform on the relative abundances of each species. Finally, extending our surveillance activities earlier in the spring may better determine the seasonal abundances of some mosquito species and the associated weather factors (e.g., *Oc. dorsalis*).

## Conclusion

We carried out 2 years of seasonal surveillance in Manitoba, Canada to explore the seasonal population dynamics and associated weather factors. The environmental conditions varied markedly between years (e.g. 2020: abnormally high rainfall concentrated over a few days; 2021: very dry), allowing us to capture a wide range of weather variables. Previous work has investigated the associations between temperature/precipitation and *Cx. tarsalis* trap counts in the Canadian Prairies [61], but to our knowledge this is the first study to explore other commonly found vector species in this region. The pervasiveness, seasonal activity, and associations with weather variables differed among species, likely due to their unique ecologies and behaviors. From our experiences, future surveillance efforts in the Canadian Prairies may benefit from using multiple trap types and a breadth of sampling sites. Placing moisture and temperature probes adjacent to each trap would improve accuracy and investigating other meteorological elements could provide further insights. Future studies aimed at associating the population dynamics of these vector species with pathogen infection rates would provide valuable information for surveillance programs. Ultimately these discrete differences among mosquito species in optimal weather conditions will influence their vector potential on an annual basis.

## Supplementary Information

**Additional file 1.** Location, coordinates, regional or rural municipality, and population size for each Western (A) and Central/Eastern (B) Manitoba mosquito trapping location.

**Additional file 2.** Trapping locations for each city/town in 2020 and 2021.

## Data Availability

Data for this study is available at: https://doi.org/10.5281/zenodo.7569133

## References

[1] Greer A, Ng V, Fisman D (2008). Climate change and infectious diseases in North America: the road ahead. Can Med Assoc J.

[2] Zhang X, Flato G, Kirchmeier-Young M, Vincent L, Wan H, Wang W, Bush E, Lemmen DS (2019). Changes in temperature and precipitation across Canada. Canada’s changing climate Report.

[3] Asgarian TS, Moosa-Kazemi SH, Sedaghat MM (2021). Impact of meteorological parameters on mosquito population abundance and distribution in a former malaria endemic area, central Iran. Heliyon.

[4] Ludwig A, Zheng H, Vrbova L, Drebot MA, Iranpour M, Lindsay LR (2019). Increased risk of endemic mosquito-borne diseases in Canada due to climate change. Can Commun Dis Rep.

[5] Trawinski PR, Mackay DS (2008). Meteorologically conditioned time-series predictions of West Nile virus vector mosquitoes. Vector Borne Zoonotic Dis.

[6] Degaetano AT (2005). Meteorological effects on adult mosquito (*Culex*) populations in metropolitan New Jersey. Int J Biometeorol.

[7] Pecoraro HL, Day HL, Reineke R, Stevens N, Withey JC, Marzluff JM, Meschke JS (2007). Climatic and landscape correlates for potential West Nile virus mosquito vectors in the Seattle region. J Vector Ecol.

[8] Walsh AS, Glass GE, Lesser CR, Curriero FC (2008). Predicting seasonal abundance of mosquitoes based on off-season meteorological conditions. Environ Ecol Stat.

[9] de Almeida Costa EAP, de Mendonça Santos EM, Correia JC, de Albuquerque CMR (2010). Impact of small variations in temperature and humidity on the reproductive activity and survival of *Aedes aegypti* (Diptera, Culicidae). Rev Bras Entomol.

[10] Panackal AA (2016). Global climate change and infectious diseases: Invasive mycoses. J Earth Sci Clim Change.

[11] Khan MA, Elhossary S, Khan IA, Al Zahrani MH, Al Zahrani FS, Al Bashri FM (2018). The impact of climatic variables with GIS application on the abundance of medically important mosquitoes (Diptera Culicidae) in Jeddah, Saudi Arabia. Int J Mosq Res..

[12] Bellone R, Failloux A-B (2020). The role of temperature in shaping mosquito-borne viruses transmission. Front Microbiol.

[13] Roiz D, Ruiz S, Soriguer R, Figuerola J (2014). Climatic effects on mosquito abundance in Mediterranean wetlands. Parasit Vectors.

[14] Buxton PA (1933). The effect of climatic conditions upon populations of insects. Trans R Soc Trop Med Hyg.

[15] Kramer LD, Hardy JL, Presser SB (1983). Effect of temperature of extrinsic incubation on the vector competence of *Culex tarsalis* for western equine encephalomyelitis virus. Am J Trop Med Hyg.

[16] Reeves WC, Hardy JL, Reisen WK, Milby MM (1994). Potential effect of global warming on mosquito-borne arboviruses. J Med Entomol.

[17] Eisen L, Monaghan AJ, Lozano-Fuentes S, Steinhoff DF, Hayden MH, Bieringer PE (2014). The impact of temperature on the bionomics of *Aedes* (*Stegomyia*) *aegypti*, with special reference to the cool geographic range margins. J Med Entomol.

[18] Ng V, Rees EE, Lindsay LR, Drebot MA, Brownstone T, Sadeghieh T, Khan SU (2019). Increased risk of exotic mosquito-borne diseases with climate change. Can Commun Dis Rep.

[19] Koloski CW, Drahun I, Cassone BJ (2021). Occurrence of the mosquito *Aedes triseriatus* (Diptera: Culicidae) beyond its most northwestern range limits in Manitoba, Canada. J Med Entomol.

[20] Grimstad PR, Haramis LD (1984). *Aedes Triseriatus* (Diptera: Culicidae) and La Crosse Virus III. enhanced oral transmission by nutrition-deprived mosquitoes. J Med Entomol.

[21] Reisen WK, Fang Y, Martinez VM (2006). Effects of temperature on the transmission of West Nile virus by *Culex tarsalis* (Diptera: Culicidae). J Med Entomol.

[22] Paz S (2015). Climate change impacts on West Nile virus transmission in a global context. Philos Trans R Soc B Biol Sci.

[23] Drakou K, Nikolaou T, Vasquez M, Petric D, Michaelakis A, Kapranas A, Papatheodoulou A, Koliou M (2020). The effect of weather variables on mosquito activity: a snapshot of the main point of entry of Cyprus. Int J Environ Res Public Health.

[24] Wijesundera MdS (1988). Malaria outbreaks in new foci in Sri Lanka. Parasitol Today.

[25] Rakotoarinia MR, Guillaume Blanchet F, Gravel D, Lapen DR, Leighton PA, Ogden NH, Ludwig A (2022). Effects of land use and weather on the presence and abundance of mosquito-borne disease vectors in a urban and agricultural landscape in Eastern Ontario, Canada. PLoS ONE.

[26] Clements AN (1992). The biology of mosquitoes: development, nutrition and reproduction.

[27] Day JF (2016). Mosquito oviposition behavior and vector control. Insects.

[28] Holmes CJ, Benoit JB (2019). Biological adaptations associated with dehydration in mosquitoes. Insects.

[29] Ogden NH, Lindsay LR, Ludwig A, Morse AP, Zheng H, Zhu H (2019). Weather-based forecasting of mosquito-borne disease outbreaks in Canada. Can Commun Dis Rep.

[30] Ripoche M, Campagna C, Ludwig A, Ogden NH, Leighton PA (2019). Short-term forecasting of daily abundance of West Nile virus vectors *Culex pipiens-restuans* (Diptera: Culicidae) and *Aedes vexans* based on weather conditions in Southern Québec (Canada). J Med Entomol.

[31] Beadle K. *Coquillettidia perturbans*. 2019. https://animaldiversity.org/accounts/Coquillettidia_perturbans/#41993238-0262-11E6-B4EB-A820662394EA. Accessed 25 Apr 2022.

[32] Lim AY, Cheong HK, Chung Y, Sim K, Kim JH (2021). Mosquito abundance in relation to extremely high temperatures in urban and rural areas of Incheon Metropolitan City, South Korea from 2015 to 2020: an observational study. Parasit Vectors.

[33] Wang X, Wang J, Russell C, Proctor P, Bello R, Higuchi K, Zhu H (2014). Clustering of the abundance of West Nile virus vector mosquitoes in Peel Region, Ontario, Canada. Environ Ecol Stat.

[34] Paaijmans KP, Wandago MO, Githeko AK, Takken W, Vulule J (2007). Unexpected high losses of *Anopheles gambiae* larvae due to rainfall. PLoS ONE.

[35] Koenraadt CJM, Harrington LC (2009). Flushing effect of rain on container-inhabiting mosquitoes *Aedes aegypti* and *Culex pipiens* (Diptera: Culicidae). J Med Entomol.

[36] Benedum CM, Seidahmed OME, Eltahir EAB, Markuzon N (2018). Statistical modeling of the effect of rainfall flushing on dengue transmission in Singapore. PLoS Negl Trop Dis.

[37] Lebl K, Brugger K, Rubel F (2013). Predicting *Culex pipiens/restuans* population dynamics by interval lagged weather data. Parasit Vectors.

[38] Schmidt CA, Comeau G, Monaghan AJ, Williamson DJ, Ernst KC (2018). Effects of desiccation stress on adult female longevity in *Aedes aegypti* and *Ae. albopictus *(Diptera: Culicidae): results of a systematic review and pooled survival analysis. Parasit Vectors.

[39] Drebot M (2015). Emerging mosquito-borne bunyaviruses in Canada. Can Commun Dis Rep.

[40] Weissmann M. Mosquito of the month: *Aedes vexans*—the inland floodwater mosquito. 2016. http://www.vdci.net/blog/mosquito-of-the-month-aedes-vexans-the-inland-floodwater-mosquito. Accessed 21 May 2022.

[41] O’Donnell KL, Bixby MA, Morin KJ, Bradley DS, Vaughan JA (2017). Potential of a northern population of *Aedes vexans* (Diptera: Culicidae) to transmit Zika virus. J Med Entomol.

[42] Parry R, Naccache F, Ndiaye EH, Fall G, Castelli I, Lühken R (2020). Identification and RNAi profile of a novel iflavirus infecting Senegalese *Aedes vexans arabiensis* mosquitoes. Viruses.

[43] Wood DM, Dang PT, Ellis RA. The insects and arachnids of Canada, Part 6. The mosquitoes of Canada: Diptera: Culicidae. Agriculture Canada Research Branch; 1979.

[44] Andreadis TG, Anderson JF, Armstrong PM, Main AJ (2008). Isolations of Jamestown Canyon virus (Bunyaviridae: Orthobunyavirus) from field-collected mosquitoes (Diptera: Culicidae) in Connecticut, USA: a ten-year analysis, 1997–2006. Vector-Borne Zoonotic Dis.

[45] Darsie RF, Ward RA (1981). Identification and geographical distribution of the mosquitoes of North America, north of Mexico. Mosq Syst.

[46] Venkatesan M, Westbrook CJ, Hauer MC, Rasgon JL (2007). Evidence for a population expansion in the West Nile virus vector *Culex tarsalis*. Mol Biol Evol.

[47] Anderson JF, Main AJ, Armstrong PM, Andreadis TG, Ferrandino FJ (2015). Arboviruses in North Dakota, 2003–2006. Am J Trop Med Hyg.

[48] Berry RL, Parsons MA, Lalonde-Weigert BJ, Lebio J, Stegmiller H, Bear GT (1986). *Aedes canadensis*, a vector of La Crosse virus (California serogroup) in Ohio. J Am Mosq Control Assoc.

[49] McMahon TJ, Galloway TD, Anderson RA (2008). Tires as larval habitats for mosquitoes (Diptera: Culicidae) in southern Manitoba, Canada. J Vector Ecol.

[50] Carpenter SJ, LaCasse WJ (1955). Mosquitoes of North America (North of Mexico).

[51] Thielman AC, Hunter FF (2007). Photographic key to the adult female mosquitoes (Diptera: Culicidae) of Canada. Can J Arthropod Identif.

[52] R Core Team (2022). R: a language and environment for statistical computing.

[53] Brooks ME, Kristensen K, van Benthem KJ, Magnusson A, Berg CW, Nielsen A, Skaug HJ, Maechler M, Bolker BM (2017). glmmTMB balances speed and flexibility among packages for zero-inflated generalized linear mixed modeling. R J.

[54] Lenth R. emmeans: estimated marginal means, aka least-squares means. R package version 1.7.0; 2021.

[55] Fox J, Weisberg S (2019). An R companion to applied regression.

[56] Hartig F. DHARMa: DHARMa: residual diagnostics for hierarchical (multi-level/mixed) regression models. R package version 0.4.4; 2021.

[57] Lüdecke D, Ben-Shachar M, Patil I, Waggoner P, Makowski D (2021). performance: an R package for assessment, comparison and testing of statistical models. J Open Source Softw.

[58] Wickham H (2016). ggplot2: elegant graphics for data analysis.

[59] Brust RA, Ellis RA (1976). Mosquito Surveys in Manitoba during 1975. Can J Public Health.

[60] Lysyk TJ (2010). Species abundance and seasonal activity of mosquitoes on cattle facilities in Southern Alberta, Canada. J Med Entomol.

[61] Chen C-C, Epp T, Jenkins E, Waldner C, Curry PS, Soos C (2013). Modeling monthly variation of *Culex tarsalis* (Diptera: Culicidae) abundance and West Nile virus infection rate in the Canadian Prairies. Int J Environ Res Public Health.

[62] Poirier LM, Berry KE (2011). New distribution information for *Coquillettidia perturbans* (Walker) (Diptera, Culicidae) in northern British Columbia. Can J Vector Ecol.

[63] Sérandour J, Willison J, Thuiller W, Ravanel P, Lempérière G, Raveton M (2010). Environmental drivers for *Coquillettidia* mosquito habitat selection: a method to highlight key field factors. Hydrobiologia.

[64] Nelms BM, Macedo PA, Kothera L, Savage HM, Reisen WK (2013). Overwintering biology of *Culex* (Diptera: Culicidae) mosquitoes in the Sacramento Valley of California. J Med Entomol.

[65] Foster AF, Walker ED. Mosquitoes. In: Medical and veterinary entomology, 3rd edition. Elsevier; 2019. p. 261–325.

[66] Milankov V, Petrić D, Vujić A, Vapa L (2009). Taxonomy, biology, genetic variability and medical importance of *Ochlerotatus caspius* (Pallas, 1771) and *O. dorsalis* (Meigen, 1830) (Diptera: Culicidae). Acta Entomol Serbica.

[67] Telford AD (1958). The pasture *Aedes* of Central and Northern California. Seasonal history. Ann Entomol Soc Am.

[68] Asigau S, Parker PG (2018). The influence of ecological factors on mosquito abundance and occurrence in Galápagos. J Vector Ecol.

[69] Zwiebela LJ, Takken W (2004). Olfactory regulation of mosquito–host interactions. Insect Biochem Mol Biol..

[70] Wigglesworth VB (1972). The principles of insect physiology. Science.

[71] Stuart T. An overview of the West Nile virus and California serogroup of vector competent mosquito species in the Northwest Territories from 2004–2018. TDTS Consulting, Government of Northwest Territories; 2020.

[72] Dodson BL, Kramer LD, Rasgon JL (2012). Effects of larval rearing temperature on immature development and West Nile virus vector competence of *Culex tarsalis*. Parasit Vectors.

[73] Dryer E. Animal Diversity Web. 2012. https://animaldiversity.org/accounts//. Accessed 25 Jan 2023.

[74] DiMenna MA, Bueno R, Parmenter RR, Norris DE, Sheyka JM (2006). Emergence of West Nile virus in mosquito (Diptera: Culicidae) communities of the New Mexico Rio Grande Valley. J Med Entomol..

[75] Madder DJ, Surgeoner GA, Helson BV (1983). Number of generations, egg production, and developmental time of *Culex pipiens* and *Culex restuans* (Diptera: Culicidae) in Southern Ontario. J Med Entomol.

[76] Alto BW, Juliano SA (2001). Precipitation and temperature effects on populations of *Aedes albopictus* (Diptera: Culicidae): implications for range expansion. J Med Entomol.

[77] Delatte H, Gimonneau G, Triboire A, Fontenille D (2009). Influence of temperature on immature development, survival, longevity, fecundity, and gonotrophic cycles of *Aedes albopictus*, vector of chikungunya and dengue in the Indian Ocean. J Med Entomol.

[78] Loetti V, Schweigmann N, Burroni N (2011). Development rates, larval survivorship and wing length of *Culex pipiens* (Diptera: Culicidae) at constant temperatures. J Nat Hist.

[79] Hwang M-J, Kim H-C, Klein TA, Chong S-T, Sim K, Chung Y, Cheong H-K (2020). Comparison of climatic factors on mosquito abundance at US Army Garrison Humphreys, Republic of Korea. PLoS ONE.

[80] Seah A, Aik J, Ng L-C (2021). Effect of meteorological factors on *Culex* mosquitoes in Singapore: a time series analysis. Int J Biometeorol.

[81] Paaijmans KP, Thomas MB (2011). The influence of mosquito resting behaviour and associated microclimate for malaria risk. Malar J.

[82] Ciota AT, Matacchiero AC, Kilpatrick AM, Kramer LD (2014). The effect of temperature on life history traits of *Culex* mosquitoes. J Med Entomol.

[83] Phanitchat T, Apiwathnasorn C, Sumroiphon S, Samung Y, Naksathit A, Thawornkuno C, Juntarajumnong W, Sungvornyothin S (2017). The influence of temperature on the developmental rate and survival of *Aedes albopictus* in Thailand. Southeast Asian J Trop Med Public Health.

[84] Epstein PR (2001). Climate change and emerging infectious diseases. Microbes Infect.

[85] Chaves LF, Hamer GL, Walker ED, Brown WM, Ruiz MO, Kitron UD (2011). Climatic variability and landscape heterogeneity impact urban mosquito diversity and vector abundance and infection. Ecosphere.

[86] Wang J, Ogden NH, Zhu H (2011). The impact of weather conditions on *Culex pipiens* and *Culex restuans* (Diptera: Culicidae) abundance: a case study in Peel Region. J Med Entomol.

[87] Chuang TW, Ionides EL, Knepper RG, Stanuszek WW, Walker ED, Wilson ML (2012). Cross-correlation map analyses show weather variation influences on mosquito abundance patterns in Saginaw County, Michigan, 1989–2005. J Med Entomol.

[88] Ho BC, Chan KL, Chan YC (1971). *Aedes aegypti* (L.) and *Aedes albopictus* (Skuse) in Singapore City. Bull World Health Organ.

[89] Toma T, Miyagi I (1990). Seasonal changes in the hatchability of *Aedes albopictus* (Diptera: Culicidae) eggs in Okinawajima, Ryukyu Archipelago, Japan. Jpn J Sanit Zool.

[90] Lourenco-de-Oliveira R, Castro MG, Braks MA, Lounibos LP (2004). The invasion of urban forest by dengue vectors in Rio de Janeiro. J Vector Ecol.

[91] Richards SL, Apperson CS, Ghosh SK, Cheshire HM, Zeichner BC (2006). Spatial analysis of *Aedes albopictus* (Diptera: Culicidae) oviposition in suburban neighbourhoods of a Piedmont community in North Carolina. J Med Entomol.

[92] Sulaiman S, Jeffery J (1986). The ecology of *Aedes albopictus* (Skuse) (Diptera: Culicidae) in a rubber estate in Malaysia. Bull Entomol Res.

[93] Toma L, Severini F, Di Luca M, Bella A, Romi R (2003). Seasonal patterns of oviposition and egg hatching rate of *Aedes albopictus* in Rome. J Am Mosq Control Assoc.

[94] Hayes RO, Maxwell EL, Mitchell CJ, Woodzick TL (1985). Detection, identification, and classification of mosquito larval habitats using remote sensing scanners in earth-orbiting satellites. Bull World Health Organ.

[95] Pratt HD, Moore CG (1986). Mites of public health importance and their control.

[96] Johnson PH, Russell RC (2019). Colonization of *Coquillettidia linealis* (Skuse) with reference to other *Coquillettidia* and *Mansonia* species. J Vector Ecol.

[97] Why AM, Walton WE (2020). Oviposition behavior of *Culex tarsalis* (Diptera: Culicidae) responding to semiochemicals associated with the western mosquitofish, *Gambusia affinis* (Cyprinodontiformes: Poecilliidae). J Med Entomol.

