# Supplementary material for: The influence of weather on the population dynamics of common mosquito vector species in the Canadian Prairies
Source: Parasit Vectors. 2023 Apr 28;16:153. doi: 10.1186/s13071-023-05760-x (PMC10148408; doi:10.1186/s13071-023-05760-x)
Supplement: Supplementary file 1 — Additional file 1. Location, coordinates, regional or rural municipality, and population size for each Western (A) and Central/Eastern (B) Manitoba mosquito trapping location. [file 13071_2023_5760_MOESM1_ESM.docx]

**Additional file S1**. Location, coordinates, regional or rural municipality, and population size for each Western (A) and Central/Eastern (B) Manitoba mosquito trapping location.

**(A)**

| **Location** | **Coordinates** | **Municipality** | **Population** |
| --- | --- | --- | --- |
| Boissevain | 49°13′50″N 100°03′30″W | Boissevain-Morton | 1,572 |
| Brandon | 49°50′54″N 099°57′00″W | Brandon | 48,859 |
| Carberry | 49°52′08″N 99°21′34″W | North Cypress-Langford | 1,738 |
| Cypress River | 49°33′22″N 99°27′12″W | Victoria | 175 |
| Killarney | 49°11′00″N 99°39′46″W | Killarney-Turtle Mountain | 2,429 |
| Shoal Lake | 50°26′16″N 100°35′28″W | Yellowhead | 714 |
| Souris | 49°37′15″N 100°15′29″W | Souris-Glenwood | 1,867 |
| Virden | 49°51′03″N 100°55′54″W | Wallace-Woodworth | 3,114 |

**(B)**

| **Location** | **Coordinates** | **Municipality** | **Population** |
| --- | --- | --- | --- |
| Winnipeg  Metropolitan region | 49.88°N 97.13°W | Winnipeg | 821,537 |
| Gimli | 50°38′01″N 96°59′24″W | Gimli | 2,246 |
| Winnipeg Beach | 50°30′21″N 096°58′27″W | Bordered by R.M. of  Gimli, St. Andrews and Dunnottar | 1,145 |
| Ste. Anne | 49°40′11″N 96°38′58″W | Ste. Anne | 2,114 |
| Portage la Prairie | 49.9723° N, 98.2903° W | Portage la Prairie | 13,304 |
| Steinbach | 49°31′33″N 96°41′02″W | Steinbach | 15,829 |
| Morris | 49°21′18″N 97°21′54″W | Morris | 1,916 |
| Altona | 49°06′16″N 97°33′45″W | Rhineland | 4,212 |
| Winkler | 49°10′54″N 97°56′23″W | Stanley | 12,660 |
