# Supplementary material for: The influence of weather on the population dynamics of common mosquito vector species in the Canadian Prairies
Source: Parasit Vectors. 2023 Apr 28;16:153. doi: 10.1186/s13071-023-05760-x (PMC10148408; doi:10.1186/s13071-023-05760-x)
Supplement: Supplementary file 2 — Additional file 2. Trapping locations for each city/town in 2020 and 2021. [file 13071_2023_5760_MOESM2_ESM.docx]

| **City/Town** | **Year** | **Trapping Locations** |
| --- | --- | --- |
| Shoal Lake | 2020 | 2 |
| Virden |  | 2 |
| Souris |  | 2 |
| Carberry |  | 2 |
| Cypress River |  | 2 |
| Brandon |  | 10 |
| Boissevain |  | 2 |
| Killarney |  | 2 |
| Shoal Lake | 2021 | 1 |
| Virden |  | 1 |
| Souris |  | 1 |
| Carberry |  | 1 |
| Cypress River |  | 1 |
| Brandon |  | 1 |
| Boissevain |  | 1 |
| Killarney |  | 1 |
| Altona* | 2020 |  |
| Gimli* |  |  |
| Morris* |  |  |
| Portage la Prairie* |  |  |
| Ste. Anne* |  |  |
| Steinbach * |  |  |
| Winkler* |  |  |
| Winnipeg Beach* |  |  |
| Winnipeg * |  |  |

**Additional file S2**. Trapping locations for each city/town in 2020 and 2021.

*Traps were managed by external party; we received selected catches throughout the season.
